# Stem cells and ageing in the blood: an interview with Margaret (Peggy) Goodell

**DOI:** 10.1242/dmm.052784

**Published:** 2025-12-17

**Authors:** Margaret (Peggy) Goodell

**Affiliations:** Molecular and Cellular Biology Department, Baylor College of Medicine, 1 Baylor Plaza, BCM130, Houston, TX 77030, USA

## Abstract

Margaret (Peggy) Goodell has significantly advanced our understanding of haematopoietic stem cells – the stem cells that develop into different types of specialised blood cells. She is Professor and Chair of the Department of Molecular and Cellular Biology at Baylor College of Medicine in Houston, TX, USA. Peggy completed her PhD at the University of Cambridge, UK, before returning to the USA to carry out postdoctoral work at the Whitehead Institute for Biomedical Research, in Cambridge, MA, where she pioneered a novel technique for isolating haematopoietic stem cells, known as the ‘side population’ method. She then joined Baylor College of Medicine in 1997 to start her own lab and has stayed there since. Peggy's research focuses on regulatory mechanisms in haematopoietic stem cells and how these go awry during ageing and disease. The significance of her research has been recognised by multiple prestigious awards, including the Tobias Award from the International Society for Stem Cell Research and the Dameshek Prize from the American Society of Hematology. In this ‘A Model for Life’ interview, we discuss Peggy's impressive career path, the parallels between ageing and cancer in the blood, and the lessons we can learn from stem cell biology to understand and mitigate disease.


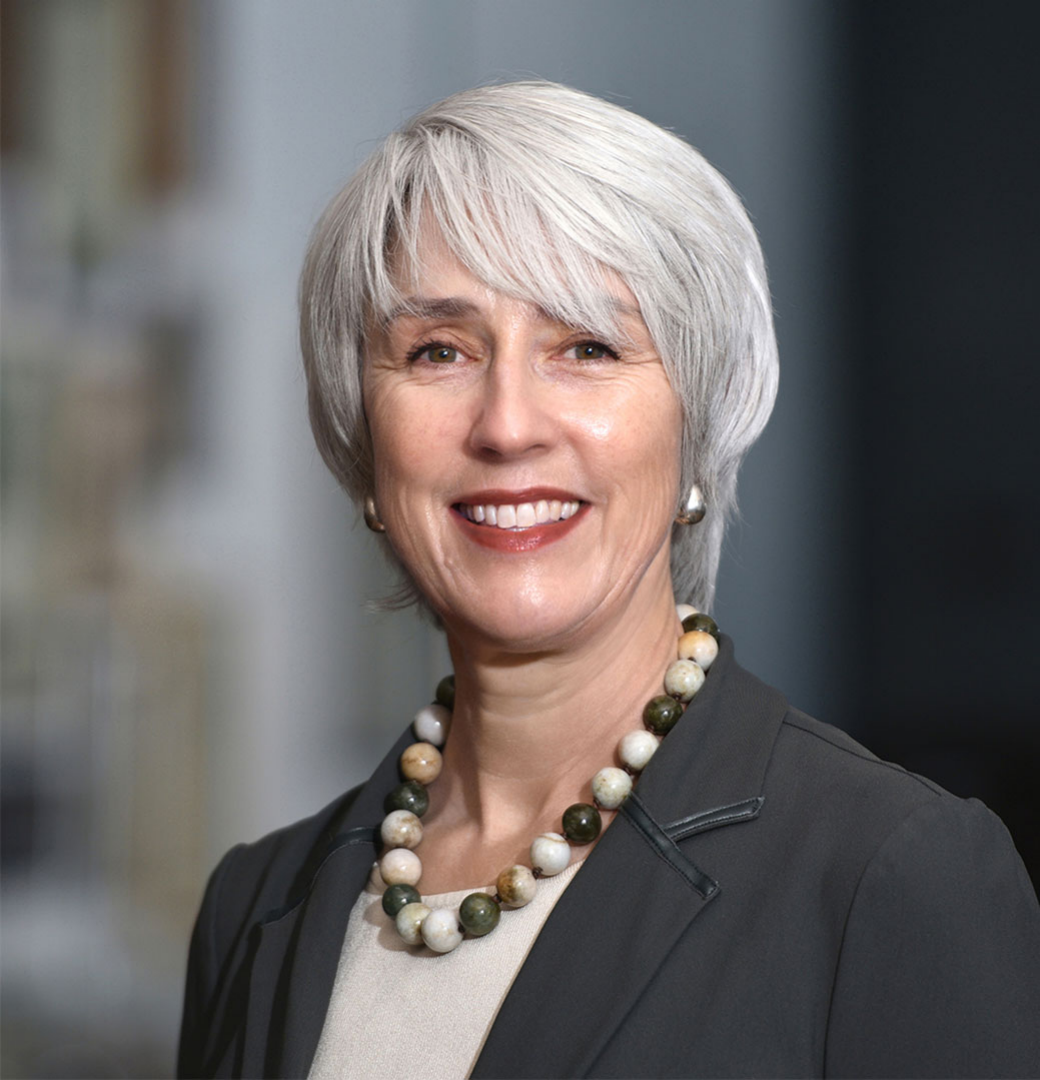


**Margaret (Peggy) Goodell**

**Let's start at the beginning: how did you first become interested in science?**

I was always very curious about the natural world as I grew up in the countryside surrounded by nature. My mother was a bird watcher, and my grandmother was always trying to encourage my interests by sending me books on science that were age appropriate. So, it started from an early age, and I got more and more interested over time.

**You're from the USA but moved to the UK during your undergraduate degree and then stayed for your PhD. How did you find living and studying abroad at this stage in your career?**

It's a bit of an unusual trajectory. I started my undergraduate studies at Wesleyan University in Connecticut, USA. I really wanted to do a semester abroad so I found a programme I could join at Imperial College London, UK. I really enjoyed the way that the studies in molecular biology were structured there, so I decided to stay at Imperial College and get my undergraduate degree there. Then, when I looked at PhD programmes, I ultimately decided to stay in the UK and go to Cambridge. Having grown up in a small town, moving to London was just fabulous. It gave me an opportunity to travel a lot around Europe, so, personally, it was a great period of growth. Science was done a little bit differently in those days between the USA and UK, too – it still is. It was a good experience and good training to see those differences. After my PhD, I figured, as an American, it was time to go back to learn how to do science again in the USA, so I came back for my postdoc and have stayed since.

**You have made significant contributions to the hematopoietic stem cell field. How did you come to work in this area?**

During my PhD, I started working with embryonic stem cells, which was before other people really knew what they were. We got a culture from Martin Evans' lab, which was very, very exciting. I would say we didn't really anticipate where the field would go at the time; we just knew that it was exciting. From this work, I realised that stem cells were very interesting and had all this potential, so I looked around at what other stem cell systems were feasible to work on. This got me interested in haematopoietic stem cells – at the time, there were not very many labs working on them. I went to a lab that was interested in haematopoietic stem cells as a vehicle for gene therapy. In that lab, there were always one or two people working on the basic biology of stem cells, so that's what I picked up.

**What research discoveries have you found most exciting during your career?**

It's funny, many people think it must be so exciting to be a scientist because they envision ‘discovering’ new things every day, when really most of the work is fairly routine. But this steady state is punctuated by a handful of very exciting moments – there's a term in evolution, ‘punctuated equilibrium’, that I feel describes a scientific career quite well. There are three main discoveries in my career that particularly stand out to me. When I discovered the side population as a method to purify hematopoietic stem cells during my postdoc (Goodell et al., 1996), this was a total epiphany. I remember sitting at the flow cytometer late one evening analysing data and seeing this population of cells and immediately realising the implications. Then I had to test it to prove the finding, but it was a surprising and exciting observation that was very impactful for my career.

A few years later in my own lab, we had been profiling gene expression in haematopoietic stem cells, and we saw lots of inflammatory genes upregulated, particularly interferon response genes, when stem cells were stimulated to regenerate. I thought this was interesting, and we reported it in an initial draft of a manuscript (Venezia et al., 2004). The reviewers hated it. They said we must have had an infection in the animal house to cause all that inflammation in the stem cells, and they assumed the observation was wrong. I was confident the data were sound and decided to dig deeper to understand what it meant. We followed that thread and conducted more experiments on this topic, publishing several follow-up papers including one in *Nature* (Baldridge et al., 2010). Now the idea that stem cells respond to inflammation, interferons and infection is everywhere and has become a foundational tenet of the field.

The third project that comes to mind is the discovery of the role of DNA methyl transferase 3A (DNMT3A) in haematopoietic stem cells (Challen et al., 2011). I remember very clearly discussing the data with the postdoc who'd done the experiments. Through these discussions, we started looking at the data in a different way, and, finally, after summarising it in a particular graph, we realised how impactful and important the observation was. When we knocked out *DNMT3A* in the stem cells, it really inhibited their differentiation and instead caused them to make more stem cells. At the time, there was no evidence in humans suggesting that it had any role in this context whatsoever, but these data showed just how important it was for the stem cells in their decision to differentiate or not.

… because the haematopoietic system bathes all tissues, these ageing-associated signals are conveyed everywhere in the body

**How are haematopoietic stem cells impacted by ageing and what are the implications of this for the body?**

When we started working on ageing and hematopoietic stem cells, many people thought that the stem cells would be protected from ageing, acting as some sort of ‘fountain of youth’. But when we looked at their gene expression profiles and molecular markers, they were ageing just like everything else (Chambers et al., 2007; Sun et al., 2014). They became more inflamed, and they didn't differentiate as well as young stem cells and had many markers of general ageing. For example, during our gene expression profiling, this marker gene came up called clusterin. This is also upregulated in Alzheimer's disease, aged muscle and many other tissues, so I realised it was just a general ageing marker and nothing special about haematopoietic stem cells, or even stem cell ageing. Understanding this has helped us realise that when those stem cells differentiate – depending on how much their downstream cells can rejuvenate – they are propagating that inflamed, aged state to all of the cells they give rise to. Then, because the haematopoietic system bathes all tissues, these ageing-associated signals are conveyed everywhere in the body. This has made us think beyond what the stem cells themselves are doing to investigate the crosstalk between the blood and whole-organism physiology.

**You have done a lot of work on clonal haematopoiesis, a condition associated with stem cell ageing in the blood. Please could you tell us more about this condition?**

I find clonal haematopoiesis fascinating. It was really mind blowing when it was discovered. Everybody thought that the haematopoietic stem cell pool was fairly uniform in its activity, so the concept that stem cells are always acquiring mutations that drive selection for specific clonal subsets was quite mind blowing in the field. What's really interesting is that all of those mutations and their ability to drive clonal expansion of stem cells act like a mutational screen that highlights genes that are important for the stem cells. *DNMT3A* turned out to be one of these key driver mutation for clonal haematopoiesis, but there were other genes like protein phosphatase, Mg^2+^/Mn^2+^ dependent 1D (*PPM1D*) that nobody was studying in blood. When I looked it up, I realised that people worked on PPM1D in solid tumours and that it regulates the tumour suppressor p53. Clonal haematopoiesis helped us find this gene of interest and has been a real revolution in the field. It's a huge feature of ageing, particularly in humans. These clonal haematopoiesis mutations also drive effects like inflammation and impaired differentiation that characterise the ageing phenotype.

There are so many fundamental mechanisms of stem cell biology that are subverted in malignancy

**Are there similarities between ageing and cancer in the blood?**

In humans with clonal haematopoiesis mutations, the haematopoietic stem cells are less efficient in their capacity to differentiate. In fact, a lot of haematological malignancies are, to some extent, diseases of blocked differentiation – some of the mechanisms converge. You get *DNMT3A* mutations in acute myeloid leukaemia and some lymphoid leukaemias. *DNMT3A* mutations are also the biggest single driver of clonal hematopoiesis. There are other mechanisms that can block differentiation as well, but this block is a common theme between ageing and cancer in the haematopoietic system. I think inflammation is also becoming a key theme. It clearly plays a role in ageing in haematopoietic stem cells but also seems to drive some aspects of malignancy, although it's not completely understood how.

**How can understanding these processes lead to new therapeutic developments?**

Understanding the mechanisms behind this blocked differentiation is key. For example, with *DNMT3A* mutations, can we improve the functionality of DNMT3A – or other aspects of the mechanism – to overcome the block and allow differentiation? There are other proteins that we're working on that also play a role in promoting or inhibiting differentiation when they're mutated, so having a thorough understanding of those processes is really important. There are so many fundamental mechanisms of stem cell biology that are subverted in malignancy. Another example is the homeobox (HOX) genes, which are important during development. *HOXA9* and *HOXA10* are among the most highly expressed genes in hematopoietic stem cells. One type of acute myeloid leukaemia that we study massively upregulates those HOX genes. They are important for promoting the self-renewal of the cancer, just like they're important for allowing the self-renewal of the stem cells. So, if we can understand how to turn these genes off to allow the stem cells to differentiate, this could improve outcomes.

**How important do you feel it is to strike a balance between translational research and basic science that focuses on fundamental mechanisms?**

I think you need both to make the advances. Many times, even if somebody stumbles on a drug that might work in a cancer, those malignancies often become resistant. Understanding why this might have happened usually comes from looking at the basic science literature to figure out what the next steps are. I really think that we need the range of work in both the basic and the more applied areas to continue to advance therapeutic modalities.

**Your work on haematopoiesis involves both mouse and human systems. What are the most important things to consider when choosing a model system to work with?**

At the end of the day, you ideally want the system that is going to best answer your question. Even though I'm a basic scientist, I am still driven by questions that affect human health. If I'm studying mouse haematopoietic stem cells, I want to make sure that the pathways I'm studying are relevant to human haematopoietic stem cells, as this is personally what drives me. This means I might have to go and check that a gene I'm studying in mouse is actually expressed in the same way in human cells. Sometimes we can do that *in silico*, or we can do it by studying the human stem cells themselves. Certainly, there are cases where you really can't get at the human question if you are studying it in the mouse, but we can't manipulate the human cells as easily. There are a lot of things that you can do *in vitro*, but it doesn't really recapitulate the whole *in vivo* environment. So, we do both: we use human cells *in vitro*, we transplant them into mice as xenografts, and we do a lot of mouse work. Going back and forth testing your hypothesis in both systems is really valuable.

**What technology or development do you think is going to have the most impact on the field going forwards?**

I really do think the field is at an inflection point for a number of reasons. First of all, sequencing is still getting cheaper and cheaper every day – it blows my mind. People are inevitably going to be sequencing more, not just to look for DNA mutations, but also to look at gene expression differences, increasingly using single-cell technologies. Then we need to be able to analyse all these data. The AI revolution is going to make it easier to analyse these massive datasets and even to design experiments that are optimised for the AI algorithms to crunch through. I think this is going to be incredibly important. Developments like CRISPR and being able to expand haematopoietic stem cells *in vitro* are also changing things, and I think it's a combination of these advances coming together that is really transforming the field.

**You have mentored many junior scientists during your career. What approach do you take to mentorship?**

I've been really lucky to have great trainees. At the beginning of your career, you just want to publish your first couple of papers and make an impact with some discoveries, and what you need evolves over time. You still want to have that excitement about the science, but now I'm much more satisfied when I see my team achieve something. Over the years, my mentorship style has certainly evolved a little, but I try to really identify what each person wants out of their career. If they're not sure, I try to help them figure it out. Not everybody wants to become a PI: they might want to teach, or be a writer or something else. I want to help them get to where they want to be, rather than impose my own ideas of what I think they should want. Trainees are often young when they're in your lab and at a stage in life where they're trying to set themselves up for the next phase. This brings its own ups and downs and can be very challenging, so I take a lot of care with supporting them. Many of them are living abroad a long way from home and don't see their families often. I wouldn't say I try to be a surrogate parent, but I've realised that they look to me for guidance, so I try to take that very seriously and be a good guide, not just a boss.

I've realised that [my trainees] look to me for guidance, so I try to take that very seriously and be a good guide, not just a boss

**Finally, what do you like to do outside of your research?**

I have three young adult daughters. One of them is still at university, so they're still very much in and out of our lives. Giving them guidance and helping them on their paths still keeps us very busy. We have four pets – a dog and three cats – so on a day-to-day basis, they also take a lot of care and attention. My husband and I like to travel, so we do try to go to some far-flung places to see different parts of life, with and without our kids. I also like to play music, and I've been able to play my guitar with other scientists at a conference in Japan – though I would say it was far more intimidating being the entertainment than giving a talk at the conference!
